# A Single Injection with Sustained-Release Microspheres and a Prime-Boost Injection of Bovine Serum Albumin Elicit the Same IgG Antibody Response in Mice

**DOI:** 10.3390/pharmaceutics15020676

**Published:** 2023-02-16

**Authors:** Renée S. van der Kooij, Martin Beukema, Anke L. W. Huckriede, Johan Zuidema, Rob Steendam, Henderik W. Frijlink, Wouter L. J. Hinrichs

**Affiliations:** 1 Groningen Research Institute of Pharmacy, Department of Pharmaceutical Technology and Biopharmacy, University of Groningen, Antonius Deusinglaan 1, 9713 AV Groningen, The Netherlands; 2 Department of Medical Microbiology and Infection Prevention, University Medical Center Groningen, University of Groningen, Antonius Deusinglaan 1, 9713 AV Groningen, The Netherlands; 3 InnoCore Pharmaceuticals, L.J. Zielstraweg 1, 9713 GX Groningen, The Netherlands

**Keywords:** bovine serum albumin, immune response, monolithic microspheres, multi-block copolymer, single-injection vaccine, sustained release

## Abstract

Although vaccination is still considered to be the cornerstone of public health care, the increase in vaccination coverage has stagnated for many diseases. Most of these vaccines require two or three doses to be administered across several months or years. Single-injection vaccine formulations are an effective method to overcome the logistical barrier to immunization that is posed by these multiple-injection schedules. Here, we developed subcutaneously (s.c.) injectable microspheres with a sustained release of the model antigen bovine serum albumin (BSA). The microspheres were composed of blends of two novel biodegradable multi-block copolymers consisting of amorphous, hydrophilic poly(ε-caprolactone)-poly(ethylene glycol)-poly(ε-caprolactone) (PCL-PEG-PCL) blocks and semi-crystalline poly(dioxanone) (PDO) blocks of different block sizes. In vitro studies demonstrated that the release of BSA could be tailored over a period of approximately four to nine weeks by changing the blend ratio of both polymers. Moreover, it was found that BSA remained structurally intact during release. Microspheres exhibiting sustained release of BSA for six weeks were selected for the in vivo study in mice. The induced BSA-specific IgG antibody titers increased up to four weeks after administration and were of the same magnitude as found in mice that received a priming and a booster dose of BSA in phosphate-buffered saline (PBS). Determination of the BSA concentration in plasma showed that in vivo release probably took place up to at least four weeks, although plasma concentrations peaked already one week after administration. The sustained-release microspheres might be a viable alternative to the conventional prime-boost immunization schedule, but a clinically relevant antigen should be incorporated to assess the full potential of these microspheres in practice.

## 1. Introduction

Although vaccination is one of the most successful medical interventions in history, coverage has not improved over the last decade for several diseases. In 2021, 18.2 million infants worldwide remained unvaccinated with the three-dose diphtheria-tetanus-pertussis (DTP3) vaccine and an additional 6.8 million only received an initial dose. This highlights a lack of access to immunization services, which is especially a problem in low- and middle-income countries [1,2]. To improve global vaccination coverage, the World Health Organization set up the Immunization Agenda 2030, with one of the objectives being the development of new vaccines, technologies, and improved products [3]. An example of an improved vaccine product is a single-injection vaccine formulation, such as a microsphere-based formulation, for vaccines that normally require multiple doses [4,5]. With this technology, the problem of the 6.8 million infants that were only partially vaccinated with the DTP3 vaccine could, for instance, be solved.

In a previous study, we developed polymeric core-shell microspheres that released the model antigen bovine serum albumin (BSA) after a lag time of three to seven weeks [6]. By co-injecting these microspheres together with a solution of BSA, a pulsatile release profile could potentially be obtained that mimics the current prime-boost immunization schedule with multiple doses at specific time intervals. Incorporation of a clinically used antigen into such a pulsatile-release formulation might result in a prolonged immunological response after only a single administration, thereby eliminating the need for booster injections. Although such pulsatile-release formulations that mimic the prime-boost immunization schedule are known to be safe and effective [4,7,8], alternative antigen release kinetics, such as sustained release, have proven to induce strong immune responses as well [9,10,11]. Moreover, sustained-release formulations are often easier to develop and manufacture and cause fewer side effects than pulsatile-release formulations [12]. As only low levels of antigen are generated upon release from the formulation, there is a limited amount of antigen systemically available during the entire period of release. It is, therefore, worthwhile to investigate the immunological response to such a formulation. In addition, sustained release more closely resembles a natural infection, because the immune system is continuously exposed to an increasing level of antigens during the course of the infection, which is usually several days or weeks [13]. The majority of the single-injection vaccine formulations described in the literature are based on the biocompatible and biodegradable polymer poly(DL-lactide-*co*-glycolide) (PLGA) [4,9]. This polymer has the advantage of being the most extensively investigated polymer in the field of controlled release and has tunable release kinetics [14]. Hydrolytic degradation of PLGA, however, might lead to accumulation of the acidic degradation products lactic acid and glycolic acid, resulting in a pH drop within the microspheres. This might affect the structural integrity and lead to the incomplete release of the incorporated (proteinaceous) antigen [15,16,17]. Hence, alternative polymers enabling release that is mainly diffusion-controlled are highly desired, as the development of an acidic microclimate is prevented.

In this study, injectable sustained-release microspheres were developed that could serve as a single-injection vaccine formulation. These monolithic microspheres consisted of biodegradable multi-block copolymers in which BSA was incorporated. These phase-separated multi-block copolymers were composed of amorphous, hydrophilic poly(ε-caprolactone)-poly(ethylene glycol)-poly(ε-caprolactone) (PCL-PEG-PCL) blocks and semi-crystalline poly(dioxanone) (PDO) blocks. Such PEG-based polymers swell when brought into an aqueous environment, thereby allowing the gradual release of the model antigen by diffusion and avoiding the accumulation of acidic degradation products [18,19,20]. An acidic microclimate is, therefore, not formed, in contrast to PLGA-based systems. This, altogether, could allow for sustained release of structurally intact BSA over several weeks. The two multi-block copolymers used in this study differed in the weight ratio of the amorphous and semi-crystalline block, the PEG molecular weight, and the total weight fraction of PEG. We hypothesized that the release duration could be tailored by varying the blend ratio of the polymers. The BSA-loaded microspheres that most closely resembled the target in vitro release profile, that is, a linear or near-linear release over four to six weeks, were subcutaneously (s.c.) administered in mice as an in vivo proof-of-concept study. The induced BSA-specific IgG antibody responses for up to eight weeks and the BSA plasma concentration for up to four weeks were measured to determine whether the microspheres could serve as an alternative to the conventional prime-boost immunization schedule.

## 2. Materials and Methods

### 2.1. Materials

*p*-Dioxanone was obtained from HBCChem, Inc. (San Carlos, CA, USA). Anhydrous 1,4-butanediol (BDO), ε-caprolactone, and PEG with a molecular weight of 1000 g/mol (PEG_1000_) and 3000 g/mol (PEG_3000_) were purchased from Thermo Fisher Scientific (Waltham, MA, USA). Stannous octoate was purchased from Sigma-Aldrich (Zwijndrecht, The Netherlands). 1,4-Butanediisocyanate (BDI) and acetonitrile were obtained from Actu-All Chemicals B.V. (Oss, The Netherlands). Polyvinyl alcohol (PVA; 5-88 EMPROVE^®^, 85–89% hydrolyzed), hydrogen peroxide, and sulfuric acid were purchased from Merck (Darmstadt, Germany). Sodium azide, Tween 20, dichloromethane (DCM), dimethyl sulfoxide (DMSO), and octane were purchased from Thermo Fisher Scientific (Waltham, MA, USA). BSA, sodium chloride (NaCl), sodium dodecyl sulfate (SDS), and *o*-phenylenediamine dihydrochloride (OPD) tablets were obtained from Sigma-Aldrich (St. Louis, MO, USA). Sodium hydroxide was obtained from VWR International Ltd. (Leicestershire, UK). Sodium carboxymethyl cellulose (CMC; Blanose^TM^ 7HF PH) was purchased from Ashland (Covington, KY, USA). BSA sample diluent was from Cygnus Technologies (Southport, NC, USA), and horseradish peroxidase (HRP)-linked goat anti-mouse IgG antibody (1 mg/mL) was from Southern Biotech (Birmingham, AL, USA). For the phosphate-perchlorate buffer and the in vitro release medium, sodium dihydrogen phosphate dihydrate (NaH_2_PO_4_·2H_2_O) and disodium hydrogen phosphate (Na_2_HPO_4_) were purchased from Thermo Fisher Scientific (Waltham, MA, USA) and sodium perchlorate monohydrate (NaClO_4_·H_2_O) from VWR International Ltd. (EMSURE^®^, Leicestershire, UK). For the carbonate-bicarbonate buffer, sodium carbonate (Na_2_CO_3_) and sodium bicarbonate (NaHCO_3_) were obtained from Merck (Darmstadt, Germany). For the BSA-specific IgG antibody ELISA, NaCl, potassium dihydrogen phosphate (KH_2_PO_4_), and Na_2_HPO_4_ were purchased from Merck (Darmstadt, Germany), sodium dihydrogen phosphate (NaH_2_PO_4_) from VWR International Ltd. (EMSURE^®^, Leicestershire, UK), and Tween 20 from Sigma-Aldrich (St. Louis, MO, USA). Gibco^TM^ sterile-filtered 1× phosphate-buffered saline (PBS; 155 mM NaCl, 1.06 mM KH_2_PO_4_, 2.97 mM Na_2_HPO_4_·7H_2_O, pH 7.4) was purchased from Thermo Fisher Scientific (Waltham, MA, USA). This PBS was used for all experiments, unless otherwise stated. Sterile 10× PBS (1.5 M NaCl, 20 mM KH_2_PO_4_, 80 mM NaH_2_PO_4_, 30 mM KCl, pH 7.4) was obtained from VWR International Ltd. (Leicestershire, UK). Ultrapure water with a resistivity of 18.2 MΩ was obtained from a Millipore Milli-Q Integral 3 (A10) purification system and used for all experiments.

### 2.2. Polymer Synthesis and Characterization

Poly(ether ester urethane) multi-block copolymers composed of hydrophilic PCL-PEG-PCL and semi-crystalline PDO prepolymer blocks were synthesized and characterized using similar procedures as previously described [18,20].

PDO prepolymer with a target molecular weight of approximately 2800 g/mol was prepared of 356.5 or 228.3 g *p*-dioxanone in the bulk at 80 °C using 11.5 or 6.7 g anhydrous BDO to initiate the ring-opening polymerization for polymer A and B, respectively. Stannous octoate was used as a catalyst at a monomer/catalyst molar ratio of approximately 25.

[PCL-PEG_3000_-PCL] prepolymer with a target molecular weight of 4000 g/mol and [PCL-PEG_1000_-PCL] prepolymer with a target molecular weight of 2000 g/mol were synthesized similarly using 61.2 g ε-caprolactone, 183.5 g PEG_3000_, and 31.3 mg stannous octoate for [PCL-PEG_3000_-PCL], and 495.9 g ε-caprolactone, 500.9 g PEG_1000_, and 140.1 mg stannous octoate for [PCL-PEG_1000_-PCL]. The mixture was magnetically stirred at 160 °C for 69 h ([PCL-PEG_3000_-PCL]) or 73 h ([PCL-PEG_1000_-PCL]) and then cooled to room temperature.

**Figure 1:**
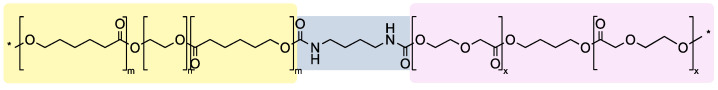
Schematic representation of the general chemical composition of the multi-block copolymers used in this study. In yellow shading: hydrophilic poly(ε-caprolactone)-poly(ethylene glycol)-poly(ε-caprolactone) (PCL-PEG-PCL) block (m: PCL, n: PEG). In blue shading: 1,4-butanediisocyanate (BDI)-based urethane linker. In purple shading: semi-crystalline 1,4-butanediol (BDO)-initiated poly(dioxanone) (PDO) block (x: PDO). The amorphous and semi-crystalline blocks are randomly distributed. The asterisks (*) indicate the possibility of having repeating subunits within the chemical structure. The two polymers used in this study, polymer A and B, differ in the weight ratio of the blocks within the copolymer (PCL-PEG-PCL block vs. PDO block), the molecular weight of PEG, and the total PEG weight fraction.

Thereafter, PDO prepolymer was chain-extended with [PCL-PEG_3000_-PCL] or [PCL-PEG_1000_-PCL] prepolymer using BDI to obtain 20[PCL-PEG_3000_-PCL]-b-80[PDO] or 50[PCL-PEG_1000_-PCL]-b-50[PDO] multi-block copolymer. To this end, approximately 300 g of [PDO] and 75 g of [PCL-PEG_3000_-PCL] were dissolved in dry 1,4-dioxane (80 °C, 30 wt-% solution), after which 20 g of BDI was added to the solution. For 50[PCL-PEG_1000_-PCL]-b-50[PDO], 189.3 g of [PDO] and 189.2 g of [PCL-PEG_1000_-PCL] were dissolved in dry 1,4-dioxane (80 °C, 30 wt-% solution), after which 21.10 g of BDI was added to the solution. Then, the reaction mixture was mechanically stirred for 20 h. Finally, 1,4-dioxane was removed from the reaction mixture by precipitation and vacuum drying. A schematic representation of the composition of the multi-block copolymers is displayed in Figure 1.

The synthesized multi-block copolymers 20[PCL-PEG_3000_-PCL]-80[PDO] (polymer A) and 50[PCL-PEG_1000_-PCL]-50[PDO] (polymer B) were analyzed for chemical composition, molecular weight, intrinsic viscosity, residual 1,4-dioxane content, and thermal properties (Table 1). Of polymer A, two different batches were prepared (hereafter referred to as polymer A_1_ and A_2_) that differed slightly in their physicochemical characteristics. The caprolactate/PEG and dioxanonate/PEG molar ratios and the weight ratio of the PCL-PEG-PCL/PDO block were determined using ^1^H NMR analysis. This demonstrated that the actual composition of the multi-block copolymers was in agreement with the targeted composition. The number average molecular weight (M_n_) and the weight average molecular weight (M_w_) were determined using gel permeation chromatography, which yielded an M_n_ of 2.8 × 10^4^ g/mol and M_w_ of 4.3 × 10^4^ g/mol for polymer A_1_ and an M_n_ of 1.5 × 10^4^ g/mol and M_w_ of 4.5 × 10^4^ g/mol for polymer A_2_. The M_n_ and M_w_ of polymer B were 3.6 × 10^4^ g/mol and 6.7 × 10^4^ g/mol, respectively. The intrinsic viscosity was approximately 0.7 dL/g for polymer A and 0.73 dL/g for polymer B, as determined with an Ubbelohde viscometer. The residual 1,4-dioxane contents as determined by gas chromatography were <18 ppm, indicating successful removal of the solvent. Modulated differential scanning calorimetry (MDSC) was used to determine the thermal behavior of the multi-block copolymers. In brief, 4–8 mg of sample was heated from −85 to 120 °C at a rate of 2 °C/min and a modulation amplitude of 0.42 °C/80 s. The glass transition temperature (T_g_, midpoint) and melting temperature (T_m_, maximum of endothermic peak) were determined using the reversed heat flow curve. Polymer A exhibited a T_g_ at approximately −15 °C, which is attributed to the amorphous PCL-PEG-PCL segments. Polymer B exhibited two T_g_ values at −57 and −23 °C, which can be ascribed to the amorphous PCL-PEG-PCL segments and the amorphous domains of the PDO block, respectively. Both multi-block copolymers exhibited a T_m_ at approximately 88 °C due to melting of the crystalline PDO segments. Polymer A exhibited another T_m_ at 34 °C, which is attributed to melting of PEG crystals.

### 2.3. Microsphere Production

BSA-loaded and placebo microspheres with a target diameter of 40 μm were produced by a membrane-assisted water-in-oil-in-water emulsion solvent extraction/evaporation method, similar to a previously described method [21]. In brief, the polymer solution was prepared by dissolving polymer A and B in the desired weight ratio in DCM to obtain a 15 wt-% solution, and filtering the solution over a 0.2 μm polytetrafluoroethylene filter. The BSA solution was prepared by dissolving BSA in PBS at a concentration of 200 mg/mL and filtering the solution over a 0.22 µm polyethersulfone filter. Subsequently, the polymer solution was homogenized with the 200 mg/mL solution of BSA in PBS (for BSA-loaded microspheres) or PBS only (for placebo microspheres) using an Ultra-Turrax^®^. The volume of BSA solution to be added was calculated to obtain a 5 wt-% target BSA loading, which resulted in a polymer solution to BSA solution ratio of 21 *v*/*v*. For the placebo microspheres, the volume of PBS to be added was calculated based on this volume ratio. The resulting primary emulsion, i.e., the dispersed phase, was injected into a continuous phase consisting of 0.4 wt-% PVA and 5 wt-% NaCl in water, by pumping the emulsion through a stainless steel membrane with 20 µm pores (20 μm × 200 μm, hydrophilic ringed stainless steel membrane; Micropore Technologies, Redcar, UK). The primary emulsion was injected at a speed of 1.3 mL/min using a Nexus 3000 syringe pump (Chemyx Inc., Stafford, TX, USA). For all formulations, a dispersed phase to continuous phase ratio of 150 *v*/*v* was used. The secondary emulsion was stirred at room temperature with a magnetic stirrer to extract and evaporate DCM. Next, the solidified microspheres were washed five times with 250 mL water and collected on a 5 μm hydrophilic polyvinylidene fluoride filter. Microspheres were freeze-dried using a Christ Alpha 2–4 LSC plus freeze-dryer (Martin Christ Gefriertrocknungsanlagen GmbH, Osterode am Harz, Germany) according to a program previously described and then stored at −20 °C [6]. The formulation and process parameters that were not mentioned above can be found in Table 2. The theoretical PEG, PCL, PDO, BDO, and BDI content of the microspheres prepared from different blend ratios of polymer A and B as determined from the in-weights is shown in Table 3.

### 2.4. Microsphere Size Analysis

For all microsphere formulations, the particle size expressed as the volume median diameter (d50) and the particle size distribution expressed as the coefficient of variation (CV) were determined with a laser diffraction particle size analyzer (Horiba LA-960, HORIBA Ltd., Kyoto, Japan). Before measurement, microspheres were dispersed in demineralized water and the obtained suspension was added to a fraction cell equipped with a magnetic stirrer to prevent sedimentation of the particles. All samples were measured immediately after addition to the cell, after which a volume-weighted size distribution plot was established according to the Fraunhofer diffraction theory. The d10 and d90 of the particle size distribution were reported as well, indicating the particle diameter at which 10% and 90% of the distribution, respectively, falls below. The CV was calculated from the d50 and the standard deviation (SD) of the distribution according to Equation (1).

(1) $$\mathrm{CV}=\frac{\mathrm{SD}}{d50}\times 100\%$$

### 2.5. Morphology of Microspheres

The surface morphology of the dried microspheres was examined using a NeoScope JCM-5000 scanning electron microscope (SEM; JEOL Ltd., Tokyo, Japan) under high vacuum and a secondary electron detector. SEM images were taken at different magnifications ranging from 50× to 1500×. The acceleration voltage was set at 10 kV, the probe current to standard, and the filament setting to long life. Prior to imaging, the microspheres were fixed onto metal sample stubs using double-sided adhesive carbon tape and sputter-coated with gold. The internal morphology was examined by mixing the microspheres with an organic solvent-free adhesive (UHU^®^ Twist & Glue Renature, Bühl, Germany). After air-drying for 2 days and cooling for 30 min at −70 °C, the samples were cut with a razor blade into five equal pieces. The obtained cross sections were imaged with SEM as described above.

### 2.6. Protein Content of Microspheres

The actual BSA loading of the microspheres was determined with the bicinchoninic acid (BCA) assay. To this end, 10 mg of microspheres was accurately weighed in triplicate in a glass tube with screw cap. Next, 1 mL of DMSO was added, and the tubes were placed in a heating block at 80 °C and vortexed to completely dissolve the polymer. After dissolution, 5 mL of 0.5 wt-% SDS in 0.05 M sodium hydroxide was added, and the tubes were placed on a roller mixer (60 rpm) overnight at room temperature to solubilize and degrade the protein. Subsequently, 100 μL of the resulting solution was pipetted into another glass tube for further analysis. BCA working reagent was prepared by mixing an alkaline BCA solution with a 4 wt-% aqueous copper(II) sulfate solution (Pierce™ BCA assay kit, Thermo Scientific, Rockford, IL, USA) in a ratio of 50 *v*/*v*, and 2 mL of the obtained working reagent was added to the tubes containing the supernatant. The tubes were vortexed and placed in a heating block at 60 °C for 30 min, after which they were cooled to room temperature and again vortexed. Samples were transferred to a plastic cuvette, and the absorbance was immediately measured at 562 nm. An eight-point calibration curve was constructed by spiking known amounts of BSA to a glass tube, and thereafter following the same procedure as described above. The calibration curve was plotted using a quadratic fit and a 1/X weighting factor to determine the actual BSA loading. The actual BSA loading was used to calculate the encapsulation efficiency (EE) according to Equation (2).

(2) $$\mathrm{EE}=\frac{\mathrm{Actual}\;\mathrm{loading}}{\mathrm{Target}\;\mathrm{loading}}\times 100\%$$

### 2.7. In Vitro Release of Microspheres

The in vitro release of BSA from the microsphere formulations was measured by accurately weighing 20 mg of microspheres in a 2 mL vial and suspending them in 1.8 mL of release medium (100 mM NaH_2_PO_4_.2H_2_O, 0.2 wt-% NaCl, 0.025 *v*/*v*% Tween 20, 0.02 wt-% sodium azide, pH 7.4, 290 mOsm/kg). In order to maintain the release medium at 37 °C, the vials were placed on a roller mixer (40 rpm) in an oven. At predetermined time intervals, the vials were placed in a centrifuge for 5 min at 4000× *g*. Next, 1.6 mL of the supernatant was collected and replaced by fresh release medium. BSA concentration in the collected release medium was determined by size-exclusion ultra-performance liquid chromatography (SE-UPLC) with fluorescence detection (λ_ex_ = 230 nm and λ_em_ = 330 nm). In brief, an ACQUITY UPLC Protein BEH SEC column (200 Å, 1.7 µm particle size, 4.6 × 150 mm, Waters, Milford, MA, USA) and a mixture of 50 mM phosphate, 0.4 M perchlorate buffer (pH 6.3) and acetonitrile (90:10, *v*/*v*) as mobile phase were used for the quantification of BSA. The liquid flow rate of this mobile phase was 0.3 mL/min. The injection volume was 5 µL and the total run time was 8.5 min. The peak areas of the main BSA peak, fragments of BSA, and aggregates of BSA were integrated at a retention time of 4.40 min, 4.67 min, and 2.00 min, respectively. An eight-point calibration curve was plotted using a quadratic fit and a 1/(X × X) weighting factor to determine the BSA concentration in the samples. For quantification of the total BSA concentration, that is, the concentration of all BSA-related compounds together, the areas of all peaks at a retention time of 2.00 to 6.00 min were integrated. As a semi-quantitative measure for the integrity of the released BSA, the BSA concentration calculated from the main BSA peak was divided by the total BSA concentration. All in vitro release curves represent the release of all BSA-related compounds together, unless otherwise stated.

### 2.8. Residual DCM Content of Microspheres

The residual DCM content in the microspheres was determined with an Agilent 6850 gas chromatograph (GC; Agilent Technologies, Santa Clara, CA, USA) equipped with a flame ionization detector, a CombiPal CTC headspace sampler, and a DB-624 column (30 m × 0.53 mm, 3 µm). As carrier gas, helium with a flow of 7 mL/min was used. The split injection mode was used with a split ratio of 1:15. The initial column temperature was 40 °C maintained for 5 min and then raised (10 °C/min) to 100 °C with a hold time of 1 min. Finally, the temperature was raised to 250 °C with 50 °C/min for 4 min. The syringe and incubation temperatures were 140 °C and 120 °C, respectively. For each formulation, 100 mg of microspheres was accurately weighed in duplicate and dissolved in 5 mL DMSO with 9.4 µg/mL octane as internal standard. Then, 2 mL of the headspace layer was injected into the GC for analysis. An eight-point calibration curve was plotted using a linear fit and a 1/X weighting factor to determine the DCM concentration from the peak area.

### 2.9. Endotoxin Level in Microspheres

The endotoxin levels in the microsphere formulations that were intended for the in vivo proof-of-concept study were determined with the Limulus amoebocyte lysate (LAL) test using a chromogenic kinetic method at a sensitivity of 0.005 EU/mL. To this end, 1 mL of DMSO was added to 50 mg of accurately weighed microspheres in duplicate, heated to 70 °C in a water bath for 1 min, and vortexed for 20 s to completely dissolve the sample. After dissolution, LAL reagent water was added to the sample (1:50 dilution), and the diluted sample and LAL/substrate reagent were added to each well of a microtiter plate. Then, the absorbance of each well was read at 405 nm and 37 °C, and this initial reading was used as the blank for the corresponding well. Subsequently, the absorbance of each well was read continuously throughout the assay. The time elapsed until the appearance of a yellow color, i.e., an increase of 0.2 absorbance units from the initial reading, was determined for each well, and this reaction time was inversely proportional to the endotoxin level in the sample. A standard curve of reaction time vs. endotoxin concentration was used to calculate the endotoxin concentration in the unknown samples. LAL reagent water was included as a negative control, and a positive product control (PPC) was prepared at a final concentration of 0.5 EU/mL. All standards and controls were assayed in duplicate as well.

### 2.10. Animal Experiments

Female CB6F1 (C57Bl/6 × BALB/c F1) mice were obtained from Charles River Laboratories (Sulzfeld, Germany). At the start of the study, the mice were eight to nine weeks old and weighed approximately 20 g. The animals were co-housed with a total number of three to six mice in individually ventilated cages and received a 12 h light/dark cycle. All animals received the rodent diet SAFE^®^ A40 (SAFE Diets, Augy, France) and tap water ad libitum. At least five days before the start of the experiment, the mice were imported to the laboratory to assure proper acclimatization. All in vivo experiments were conducted in accordance with Timeline Bioresearch AB ethical permit number 5.8.18-20232/2020.

For the in vivo proof-of-concept study, 48 mice were divided into nine groups. The treatment groups (groups A, B, and C) and positive control (plus treatment or placebo) groups (groups D to G) all contained 6 mice. The negative control groups (groups H and I) contained 3 mice. An overview of the experimental groups and the corresponding formulations used for the immunization study is given in Table 4. All formulations were administered as a 100 or 200 µL s.c. injection in the scruff of the neck under isoflurane anesthesia, and were given at day 0, unless otherwise stated. For the microspheres, 0.6 wt-% CMC solution in 10× PBS was used as the injection vehicle, whereas PBS was used for the administration of BSA solution. All treatment groups (groups A to D) were immunized with microspheres of formulation B, and the placebo group (group E) received an injection of microspheres of formulation F. The amount of microspheres to be administered was calculated from the desired dose of BSA (250, 500, or 1000 μg BSA) and the actual BSA loading of the microspheres, corrected for the percentage released in vitro after five weeks of incubation. The microspheres of groups A, B and D, and C are hereafter referred to as 250, 500, and 1000 μg BSA-microspheres, respectively. Mice of groups F and G received an injection of BSA in PBS at weeks 0 and 3, where the timing of the booster immunization was based on the experimental setup of previous immunization studies [7,22,23].

Blood samples were taken prior to administration and 1, 2, 3, 4, 6, and 8 weeks after the first administration. In total, seven blood samples were obtained from each mouse. At all time points up to six weeks, 100 µL of blood was collected in K3-EDTA tubes by sublingual bleeding and immediately placed on melting ice. During blood sampling, mice were fully conscious as no anesthesia was used, so they were gently restrained by the scruff of the neck. For the last sampling point, mice were euthanized by cervical dislocation, and all blood was collected and processed for further analysis. Then, 40 μL of plasma was prepared by centrifuging the blood samples at 1800× *g* and 4 °C for 10 min and collecting the supernatant. The obtained aliquots were placed on dry ice and eventually stored at −80 °C prior to analysis. All plasma samples were analyzed by ELISA to investigate the BSA-specific IgG antibody response of the mice. Plasma samples from weeks 1 to 4 were also tested by ELISA to determine the BSA levels.

### 2.11. ELISA for BSA-Specific IgG Antibody Titers

BSA-specific IgG antibody titers in plasma were determined by indirect ELISA. Flat-bottom high binding 96-well microplates (Greiner Bio-One, Kremsmünster, Austria) were coated overnight at 37 °C with 0.3 µg BSA (100 μL 3 µg/mL BSA solution in 0.05 M carbonate-bicarbonate buffer, pH 9.6–9.8) per well. PBS composed of 154 mM NaCl, 0.882 mM KH_2_PO_4_, and 11.4 mM Na_2_HPO_4_ and the same PBS supplemented with 0.05 *v*/*v*% Tween 20 (PBS-T) were prepared as wash solution. PBS-T was also used to remove detection antibodies. The plates were washed once with the 0.05 M carbonate–bicarbonate buffer and twice with PBS-T. Then, 1:100 dilutions of plasma samples in PBS-T were prepared and added in twofold serial dilutions to the plates, with each well containing 100 μL of a dilution. Untreated wells, i.e., wells that did not contain any plasma sample, were used to determine the plate background. After incubating the plates for 1.5 h at 37 °C, the plates were washed three times with PBS-T. Next, the plates were incubated for 1 h at 37 °C with 100 µL of a 1:5000 *v*/*v* dilution of HRP-linked goat anti-mouse IgG antibody in PBS-T to detect bound IgG antibodies. Plates were again washed three times with PBS-T and once with PBS. Then, 100 μL staining solution (20 mg OPD, 20 µL hydrogen peroxide in 100 mL 0.1 M phosphate buffer, pH 5.6) was added to each well and incubated for 30 min at room temperature shielded from light. The colorimetric reaction was stopped by adding 50 μL 2 M sulfuric acid to the wells. Absorbance was measured at 492 nm and OD values were corrected for the mean plate background. IgG antibody titers were expressed as log_2_ values of the reciprocal of the plasma sample dilution that corresponded to a corrected OD value of 0.2 at a wavelength of 492 nm, which was determined as the cut-off value. Samples with readings for the least diluted plasma lower than the cut-off value were assigned an IgG antibody titer of 5.64 log_2_, corresponding to a dilution of 1:50, which would be one dilution below the starting dilution of 1:100.

### 2.12. ELISA for BSA Quantification

Plasma BSA levels were determined with a commercial BSA ELISA kit (F030; Cygnus Technologies, Southport, NC, USA) according to the manufacturer’s instructions. Due to limited sample volume, plasma samples were diluted at least 1:2 with BSA sample diluent and analyzed only once (*n* = 1). Absorbance was measured at 450 nm using a microplate reader, and BSA concentrations were determined from a five-point calibration curve (calibration range 0.5–32 ng/mL). Some plasma samples were applied in higher dilutions of up to 1:50 to fall within the working range of the assay.

### 2.13. Statistical Analysis

All microsphere formulations (A to F, Table 2) were produced once (*n* = 1). All measurements were performed in triplicate (*n* = 3), and data were presented as mean ± SD, unless otherwise stated. The IgG titer-time data were analyzed using GraphPad Prism version 9.1.2 (La Jolla, CA, USA). The area under the IgG titer–time curve (AUC) values were obtained, and data were checked for normality using the Shapiro–Wilk test. Differences between all groups were assessed using the ordinary one-way analysis of variance (ANOVA), followed by Tukey’s multiple comparisons test for both AUC values and week 8 IgG titers. Differences between the analyzed groups were considered significant if *p* < 0.05 (* *p* < 0.05, ** *p* < 0.01).

## 3. Results and Discussion

### 3.1. Properties of Microspheres of Different Polymer Blend Ratios

**Figure 2:**
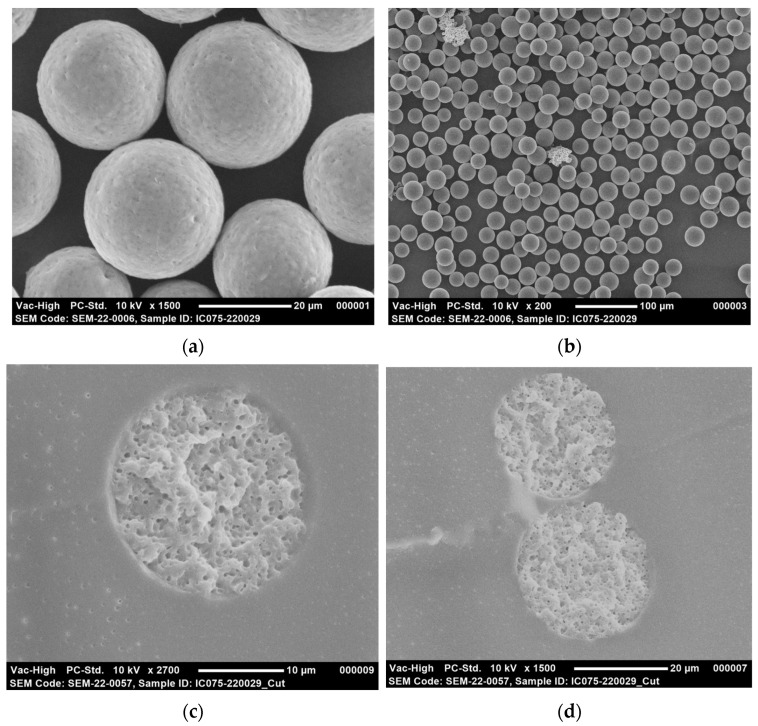
Representative scanning electron microscopy (SEM) images of microspheres loaded with 4.5 wt-% BSA (formulation B): (**a**) 1500× magnification; (**b**) 200× magnification; (**c**) Cross-sectioned microsphere at 2700× magnification; (**d**) Cross-sectioned microspheres at 1500× magnification.

Blends of polymer A and B with different weight ratios were used to prepare BSA-loaded microspheres with a 5 wt-% target loading and placebo microspheres. The polymer blend ratio and the incorporation of BSA did not seem to affect the microsphere size and size distribution, as can be seen in Table 5. All formulations had an average particle size of approximately 40 µm. This size enables parenteral administration of the microspheres through a small-gauge hypodermic needle while preventing premature uptake by cells engaging in phagocytosis [24,25]. Moreover, all microsphere formulations had a narrow particle size distribution as reflected by the relatively low CV values. This was the result of a well-defined localized shear and geometry-controlled generation of droplets in the membrane-assisted emulsification process [26]. The morphology of the microspheres was examined using SEM. Representative images of BSA-loaded microspheres composed of a 92.5:7.5 polymer blend (formulation B) are depicted in Figure 2, with Figure 2a,b revealing the surface morphology and Figure 2c,d the internal morphology. Figure 2a,b show that the microspheres had a spherical shape and a smooth and non-porous surface. As expected, images of the internal morphology (Figure 2c,d) display a monolithic matrix with numerous small pores, resulting from the fine primary emulsion used in the preparation of the microspheres. The porosity was homogeneous throughout the cross section of the particles, which implies that BSA was homogeneously distributed throughout the microspheres. A non-porous surface, small internal pores, and a homogeneous drug distribution are critical to obtaining a high EE and low initial burst release [27]. Indeed, the EE of BSA was high for all formulations (>85%, Table 5). The high EE can be attributed to the relatively high molecular weight of the polymers (M_w_ 4.3–6.7 × 10^4^ g/mol) and concentration of the polymer solution (15 wt-%), resulting in a relatively high viscosity of the polymer solution [28,29]. The high polymer solution to BSA solution ratio (21 *v*/*v*) [30] and the addition of NaCl to the continuous phase probably contributed to these high EE values as well [31].

### 3.2. In Vitro Release of BSA from Microspheres of Different Polymer Blend Ratios

**Figure 3:**
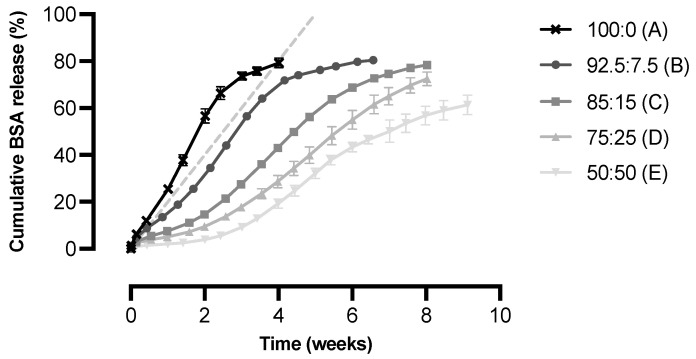
Cumulative in vitro release of BSA from microspheres composed of polymer A and polymer B in different blend ratios (*n* = 3). The cumulative release is expressed as the percentage of the total amount of BSA incorporated into the microspheres. The dashed line represents the target release profile of the microspheres with a linear release of BSA over a period of five weeks.

We investigated the suitability of a blend of multi-block copolymers A and B in obtaining microspheres with a low initial burst and linear or near-linear in vitro release of the complete BSA payload within four to six weeks. The effect of the polymer blend ratio on the in vitro release of BSA from the microspheres is presented in Figure 3. For all formulations, the initial burst release, defined as the percentage of BSA released after one day, was minimal (1 to 6%). Moreover, all release profiles showed a similar trend with an initial slow release followed by a faster release after which the release again slowed down. In particular, the microspheres composed of a relatively high percentage of polymer B exhibited such a sigmoidal release profile. The in vitro release rate was clearly influenced by the polymer blend ratio, as the release rate decreased with an increasing weight fraction of polymer B. Formulation A, which was composed of 100% of polymer A, demonstrated the highest release rate, with a cumulative release of approximately 80% after four weeks. The lowest release rate was obtained with formulation E, composed of a 50:50 polymer blend that exhibited a cumulative BSA release of only 20% after four weeks.

For bulk degrading polymers such as the polymers used in this study, drug release from the polymeric matrix is determined by the drug solubility, drug diffusion, drug load, polymer swelling, polymer degradation, or a combination of these factors [19,32]. For a hydrophilic protein with a high molecular weight, such as BSA (6.6 × 10^4^ g/mol), diffusion through the hydrated polymer matrix is dependent on the degree of swelling and degradation of the polymer matrix, as these determine the mesh size of the matrix [19]. If a mesh size larger than the protein size is reached through swelling and/or degradation, the protein will be released from the polymer matrix [19,33]. To obtain a better understanding of the in vitro release profile, it is important to determine whether the prepared microspheres exhibit diffusion- or degradation-controlled release.

In previous studies, controlled-release microspheres [20,21,34] and implants [19] were prepared from semi-crystalline, phase-separated multi-block copolymers similar to the polymers used in this study. These polymers also consisted of amorphous PCL-PEG-PCL blocks, but the semi-crystalline blocks were composed of poly(L-lactide) (PLLA) [20,21,34] or PCL [19] instead of PDO. In two of these studies, in vitro release and polymer degradation were assessed to gain insight into the release mechanisms in play. Results suggested that in vitro release was primarily driven by diffusion [19,21]. In vitro release data of several proteins were fitted into different kinetic models and in most cases, diffusion-controlled release was indicated. For the in vitro degradation studies, polymer-only microspheres [21] and implants [19] were incubated in a release medium at 37 °C, and the mass loss was determined over time. Although mass loss only gives an indication of the formation of water-soluble degradation products and degradation products that are not (yet) soluble in water will have formed as well, it does give information on the contribution of polymer degradation to the release kinetics. Only a slight mass loss was observed during the first week after incubation, which was ascribed to the preferential hydrolysis of the PEG-PCL bonds, and the subsequent dissolution and diffusion of PEG. During the remainder of the study (i.e., three [21] and nineteen [19] weeks), the sample mass hardly changed, and the molecular weight of the polymers decreased only slowly. This indicated that hydrolysis of ester bonds in the PLLA and PCL blocks was limited and that no substantial degradation had occurred within the timeframe of the degradation studies, due to slow in vitro degradation of PLLA and PCL and its copolymers. Based on the extrapolation of previously obtained data, the anticipated in vitro degradation time of PLLA-based multi-block copolymers is three to four years [35]. For the PCL-based multi-block copolymers, this is expected to be the same [36]. Therefore, the release from such multi-block copolymers was assumed to be mainly driven by other mechanisms than degradation.

In order to obtain faster degrading microspheres with a more acceptable balance between BSA release and polymer erosion, the faster degrading polymer PDO was used as the semi-crystalline block. The homopolymer has a degradation time of six months [36,37,38], and in vitro degradation of PDO-based multi-block copolymers is anticipated to be 9 to 24 months [35]. Although PDO-based multi-block copolymers degrade significantly faster than PLLA- and PCL-based multi-block copolymers, it is not expected that degradation of the semi-crystalline PDO block played a significant role in the in vitro release of BSA, as substantial degradation is unlikely to have occurred within the timeframe of the in vitro release studies (i.e., four to nine weeks) [36,37,38]. Therefore, as in previous studies, the release of BSA from the microspheres was mainly controlled by the amorphous PCL-PEG-PCL block. It is assumed that the release was partially driven by diffusion, as the high swelling degree and water solubility of the PEG blocks within the multi-block copolymer allowed the initial, diffusion-controlled release of BSA [18]. This occurred via dissolution and subsequent diffusion of the antigen through the swollen polymer matrix [19,21]. Release, however, was probably not solely diffusion-controlled but involved some degradation of the PCL-PEG-PCL block as well, which is also reflected in the sigmoidal release profile that was observed for the different microsphere formulations (Figure 3). It is assumed that ongoing degradation of the PCL-PEG-PCL blocks further increased the mesh size of the polymer matrix, which eventually accelerated the release. Especially for the formulations composed of a relatively high amount of polymer B, swelling of the polymer matrix was insufficient to cause an initial fast release of the high molecular weight BSA due to the presence of small-sized PEG blocks (Table 3). This resulted in a sort of lag phase directly after the start of the in vitro release study, after which the release rate increased. Apparently, some degradation and/or increased swelling of the polymer matrix over time was required for BSA to be released from the microspheres.

As expected, the BSA release rate from microspheres composed of polymer A and B was dependent on the polymer blend ratio, as the release rate decreased with an increasing weight fraction of polymer B (Figure 3). The slower release induced by polymer B can be explained by the fact that this polymer is less swellable and degrades slower than polymer A. As the release of BSA from the microspheres is assumed to be both diffusion- and degradation-controlled, the release rate is determined by an interplay between the PEG molecular weight, the total PEG content, the PCL content, and the PDO content of the polymer blends. The interplay between the PEG molecular weight and the total PEG content was previously described for the PLLA-based multi-block copolymers [20,21]. A comparison of polymer B with polymer A demonstrates a lower PEG molecular weight (1000 vs. 3000 g/mol), which explains the decreased release rate with an increasing weight fraction of polymer B, as PEG blocks swell due to the uptake of water. Due to the lower molecular weight of PEG in the PCL-PEG-PCL blocks, polymer B absorbs less water causing slower hydrolytic cleavage of the polymer backbone and a lower swelling degree. This eventually results in a slower release. The difference between the two polymers was also reflected in the mass loss. After incubation of polymer-only microspheres prepared from polymer A, a minor mass loss of <10% was observed after 30 days and <20% after 50 days [35]. For PDO-based multi-block copolymers comparable to polymer B, this was even less [35]. Polymer B does contain a higher total PEG content (24 vs. 15 wt-%), but this did not compensate for the PEG molecular weight. In this case, a high molecular weight of PEG is apparently more important to create a polymeric network that swells enough to allow the diffusion of the high molecular weight BSA, than a high total PEG content is for the formation of such a hydrated network. Moreover, the PCL content was higher for polymer B than for polymer A (25 vs. 5 wt-%), while the PDO content was lower (50 vs. 80 wt-%), as shown in Table 3. As PCL degrades slower than PDO, polymer B is expected to degrade slower than polymer A, resulting in a lower release rate. A higher PCL content also results in a lower swelling degree due to its hydrophobicity, thereby causing a decreased release rate [39].

**Figure 4:**
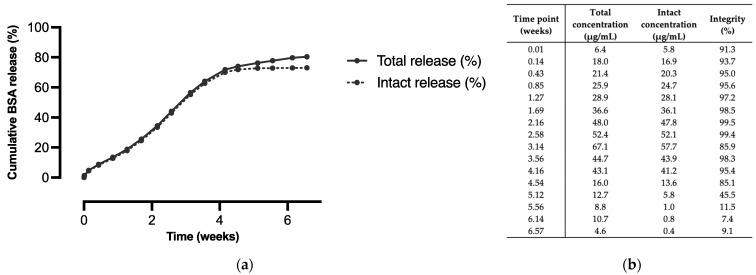
In vitro release of BSA from microspheres composed of polymer A and polymer B in the blend ratio 92.5:7.5 (formulation B, *n* = 3): (**a**) Cumulative total and intact release vs. time. (**b**) Total and intact BSA concentration and corresponding integrity of samples at each individual time point.

We aimed to develop a formulation that exhibited a continuous release of BSA for approximately four to six weeks. Microspheres prepared from a 92.5:7.5 blend of polymer A and B (formulation B) exhibited near-linear release kinetics for up to four weeks, after which the release of BSA continued in a slower fashion for another two weeks. In addition, a high cumulative release of 80% was obtained during the course of the in vitro release study. Since these microspheres best met our target in vitro release profile, this formulation was selected for the in vivo proof-of-concept study. Figure 4a presents the results of the in vitro release study with this formulation, showing both the total and the intact BSA release from the microspheres. Protein denaturation and aggregation are common problems for protein-loaded microspheres, as they are subjected to many stress factors upon incubation, such as hydration and elevated temperatures [4,25]. Although the integrity of the released BSA was not tested directly, we did measure the percentage of BSA that was released as fragments or aggregates, which indicated how well the structural integrity was maintained during incubation. During the first four weeks, the integrity of the released BSA was high (>90%, Figure 4b). Only at the end of the in vitro release study did the integrity decrease drastically, as the release mainly consisted of aggregates of BSA. These aggregates are larger than BSA itself and are, therefore, probably released more slowly. During the major part of the in vitro release study, however, aggregates and fragments of BSA were absent and the cumulative intact release was even >70%.

**Figure 5:**
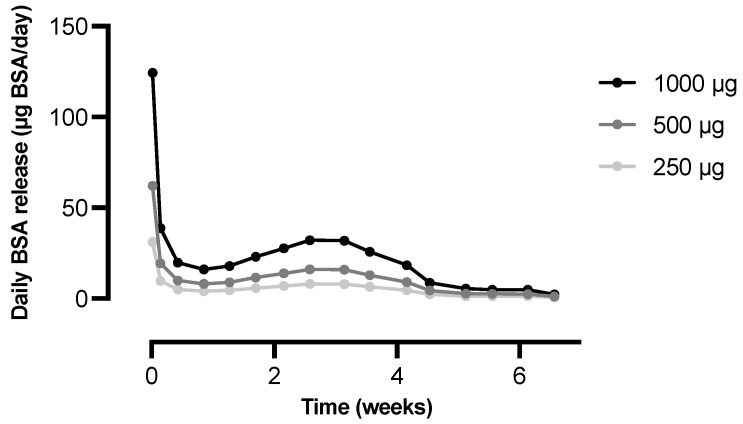
Average daily in vitro release of BSA from microspheres composed of polymer A and polymer B in the blend ratio 92.5:7.5 (formulation B, *n* = 3). The average daily in vitro release was calculated by dividing the absolute amount of BSA (in µg) that was released between two sampling points by the time between those sampling points. The different curves represent different amounts of microspheres corresponding to a total BSA content of 250, 500, or 1000 µg.

Furthermore, the average daily in vitro release from formulation B for different doses of BSA was plotted in Figure 5. A relatively high daily release is visible during the first day of incubation and especially during the first two hours due to a small initial burst release. Apart from day one, the average daily release was rather constant during the first four weeks of the release study. A slight increase in the daily release was observed up to approximately three weeks, followed by a decrease during the remaining weeks of the release study, which is typical for a sigmoidal release profile.

### 3.3. Residual DCM Content and Endotoxin Analysis of Microspheres Intended for the In Vivo Study

The residual DCM content in the microspheres of formulation B and the corresponding placebo microspheres (formulation F) was measured to determine whether the removal of the toxic organic solvent was effective. The DCM content of formulation B and F was 295 and 294 ppm, respectively, which is well below the ICH concentration limit of 600 ppm [40]. The permissible daily exposure for humans is 6 mg/day [40]. When this value is corrected for the body weight of a mouse (20 g) and for the factor that accounts for the extrapolation between both species (12), a permissible daily exposure of 28.8 µg/day is found for mice. As the highest amount of microspheres to be administered is 29.2 mg, the maximum DCM exposure will be only 8.6 µg, which is below the permissible daily exposure as well. Although there are only limited data available on DCM toxicity after parenteral administration, no increased risk of tumor development was observed in mice after oral administration of DCM doses up to 5 mg/day [41]. Therefore, no carcinogenic effects are expected from the prepared microspheres. In addition, the endotoxin level in both formulations was quantified as it is an important factor for microspheres intended for immunological studies. The LAL test confirmed that both formulations did not contain detectable levels of endotoxin (<5 EU/g microspheres). Therefore, it is not expected that any undefined immune responses will be induced by endotoxins from the microspheres. Overall, both formulations complied with all requirements for use in the in vivo proof-of-concept study.

### 3.4. IgG Antibody Response and Kinetics of Microspheres In Vivo

Based on the in vitro release results, the BSA-loaded microspheres prepared from a 92.5:7.5 blend of polymer A and B (formulation B) and the corresponding placebo microspheres (formulation F) were chosen for the in vivo proof-of-concept study in mice. The microspheres containing the model antigen BSA were s.c. injected to investigate whether the formulation could elicit a BSA-specific IgG antibody response. Different amounts of the microspheres were injected into the subcutaneous tissue to test the effect of the dose of BSA on the humoral immune response. A positive control/treatment group was included to determine whether priming with BSA in PBS could enhance the antibody response induced by the microspheres. A positive control/placebo group was included to investigate the potential adjuvant effect of the polymers. In addition, two positive controls consisting of a high- and low-dose prime-boost injection of BSA in PBS were included to compare the antibody titers induced by a prime-boost immunization schedule with the titers induced by the microspheres. Two negative controls consisting of PBS and CMC solution were included to confirm that the vehicles did not induce BSA-specific IgG antibodies. For all groups, the BSA-specific IgG antibody titers in the mouse plasma were determined over time, up to eight weeks after administration.

**Figure 6:**
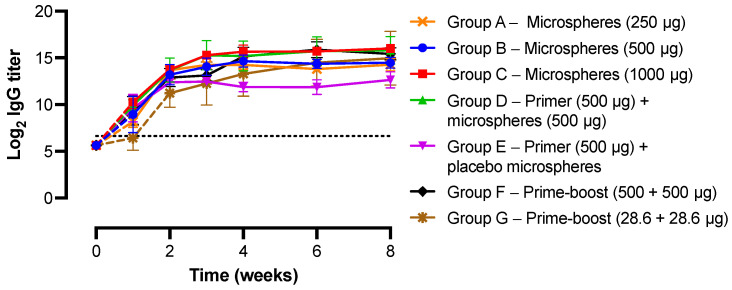
BSA-specific IgG antibody titers in mouse plasma over time after immunization with different BSA formulations (group A to G). The averages of the antibody levels measured in all mice were calculated for each group (*n* = 6 per group) and presented for all groups together. The dotted line represents the cut-off value for the IgG antibody titer, i.e., a titer of 6.64 log_2_, corresponding to the starting dilution of the plasma samples of 1:100. Values below this titer could not be measured. Samples with a reading for the least diluted plasma (i.e., 100× diluted) lower than the cut-off value were assigned an IgG antibody titer of 5.64 log_2_, corresponding to a dilution of 1:50, which would be one dilution below the starting dilution. Dashed lines were used to connect the data points with a titer of 5.64 log_2_ to the next data point. The negative control groups receiving PBS (group H) and CMC solution (group I) are not presented in this figure.

**Figure 7:**
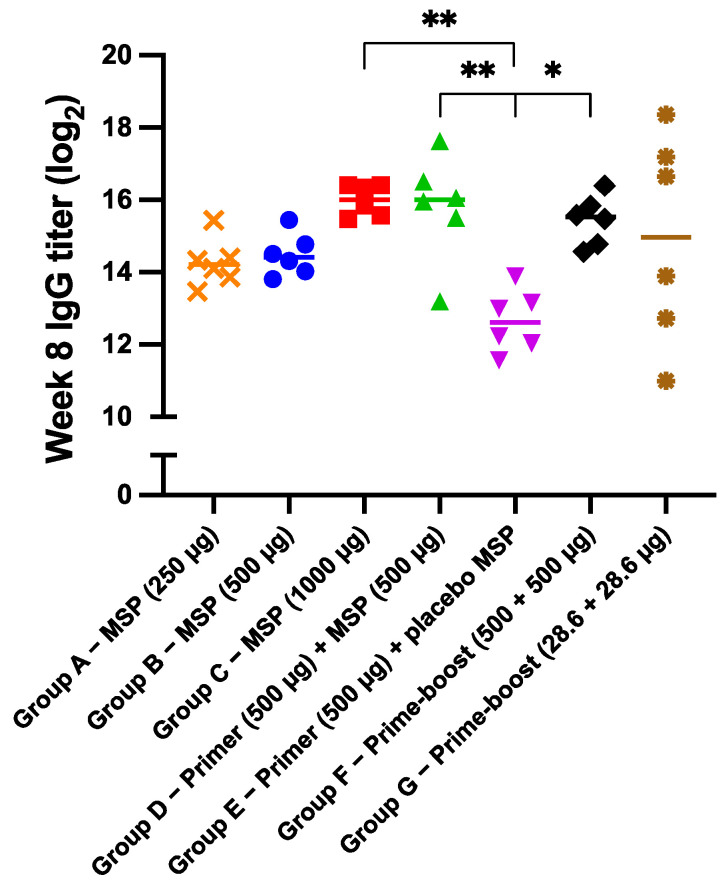
Week 8 IgG titer for each individual mouse of group A to G. Statistical comparisons between the mice of the different groups were performed using the ordinary ANOVA, followed by Tukey’s multiple comparisons test (* *p* < 0.05, ** *p* < 0.01). For clarity reasons, statistical comparison is only indicated where *p* < 0.05 (*) or *p* < 0.01 (**), and differences for all other comparisons were non-significant. The negative control groups receiving PBS (group H) and CMC solution (group I) are not presented in this figure. MSP = microspheres.

As expected, no IgG antibody response was induced after administration of the injection vehicles to the negative control groups. For the other groups, the systemic BSA-specific IgG antibody titers over time after administration of different BSA and placebo formulations are shown in Figure 6 (see Figure S1 in the Supplementary Materials for the IgG antibody titers of the individual mice). In addition, the final IgG antibody titers as measured at week 8 are shown in Figure 7. The AUC values of the antibody titer vs. time graphs from Figure S1 were calculated for each individual mouse of groups A to G as shown in Figure S2. All mice that received BSA-loaded microspheres had elevated antibody titers from week 1 onwards, which indicates that the microspheres were effective in inducing an immune response. To assess the performance of the sustained-release microspheres in relation to the conventional prime-boost immunization schedule, a high-dose prime and booster injection of BSA in PBS (500 + 500 µg BSA) were administered to mice in group F at weeks 0 and 3, respectively. As expected, antibody titers increased up to week 2, remained steady up to week 3, and again increased and stabilized at week 4 (Figure 6). In other words, the antibody titers spiked following the prime and the booster injection, which demonstrates that the conventional prime-boost immunization schedule was effective as well. Comparison of group C (1000 µg BSA-microspheres) and F (prime-boost 500 + 500 µg BSA) demonstrates that the (week 8) antibody titers as well as the AUC values of both groups were not significantly different. This shows that sustained release of BSA from the microspheres did not result in immunological tolerance toward the model antigen within the tested time frame. The induction of tolerance toward the antigen, causing the vaccine to be ineffective, has previously been related to the sustained release of the antigen from the formulation [42,43,44]. Clear evidence is, however, lacking [45], and apparently was not found in our study either. When the same total dose of BSA was given, microspheres and a prime-boost injection of BSA in PBS induced a similar IgG antibody response, so the sustained-release microspheres could be a viable alternative to the conventional prime-boost immunization schedule.

Interestingly, mice of group E that received a prime injection of 500 µg BSA in PBS and a mock immunization of placebo microspheres demonstrated a rather different immune response. Here, the antibody titers peaked already after two weeks, after which no further increase in titer was observed (Figure 6). For this group, the final IgG antibody titer at week 8 was significantly lower than that of groups C and F (*p* < 0.01 for 1000 µg BSA-microspheres and *p* < 0.05 for prime-boost 500 + 500 µg BSA). The AUC value was also significantly lower than that of group C. Even though the total administered dose of BSA was lower than for groups C and F, these results suggest that a priming dose alone is not sufficient to elicit a strong immune response over time, and that a booster injection or a continuous release of antigen is required. Although the difference was not statistically significant, the fact that the AUC value and week 8 antibody titer of group E were also lower than those of group B (500 µg BSA-microspheres), which did receive the same total dose of BSA, supports this conclusion. A single-injection vaccine formulation such as the microspheres would then have the preference over the conventional prime-boost vaccine. Apart from one outlier, mice that received both a prime injection of 500 µg BSA in PBS and 500 µg BSA-microspheres (group D, Figure 6) showed an antibody response that strongly resembled the response in group C (1000 µg BSA-microspheres), as the total administered dose of BSA was the same. Apparently, priming with BSA in PBS in addition to the sustained-release microspheres does not enhance the antibody response and, therefore, does not have a preference over the administration of microspheres only.

Furthermore, the fact that (week 8) antibody titers and AUC values were similar for groups C and F suggests that the microspheres did not possess any adjuvant activity for the encapsulated model antigen, as was observed previously for PLGA-based single-administration vaccine formulations [7,46,47]. Comparison of group E (500 µg BSA in PBS + placebo microspheres) and F (prime-boost 500 + 500 µg BSA) confirmed this suspicion, as the IgG antibody titer at week 3 was similar for both groups (12.5 ± 0.1 and 13.1 ± 0.6 log_2_, respectively).

Mice in the treatment groups (groups A to C) received different amounts of microspheres that were expected to deliver an amount of 250, 500, and 1000 µg BSA, respectively, into the subcutaneous tissue. The antibody responses measured in these groups all followed a similar trend, with an increasing titer up to four weeks, after which it leveled off (Figure 6). A clear difference between the groups is, however, visible at week 1, which demonstrates that the development of high antibody titers takes more time at lower doses. Moreover, the week 8 antibody titer in group C was 3.1- and 2.8-fold higher than in group A and B, respectively (Figure 7), although the differences were not significant (*p* > 0.05). The AUC values raised by immunization with different doses of BSA-loaded microspheres were not significantly different either (Figure S2). Possibly, the difference between the administered doses was not large enough and the doses were all relatively high, which caused only a small difference in immune response. In another study with BSA-loaded microspheres, the influence of the dose on the magnitude of the induced antibody response was more clearly visible [8]. Here, a high dose of BSA (431 µg) elicited 13- and 8-fold higher antibody titers than a low dose of BSA (i.e., 64 µg) at the first and last time point of the in vivo study, respectively. It should, however, be noted that the microspheres in this specific study displayed a pulsatile release of BSA instead of sustained release, which impedes direct comparison.

Finally, mice receiving a high- and a low-dose prime-boost injection of BSA in PBS were compared in terms of IgG antibody response. The dose of the high-dose prime-boost injection (500 + 500 µg BSA, group F) was based on the total dose of the sustained-release microspheres from group C, and the dose of the low-dose prime-boost injection (28.6 + 28.6 µg BSA, group G) was based on the average daily dose of the microspheres. All mice in the high-dose prime-boost group developed high titers of BSA-specific IgG antibodies. However, high variability in antibody titers was observed in the low-dose prime-boost group. These results are in line with a study by Guarecuco et al., where a greater variability in antibody response was observed for a low-dose than for a high-dose BSA formulation [8]. This probably indicates that in some mice of group G, the amount of antigen reaching the draining lymph nodes was sufficient for B cell activation, while in other mice this was not the case [48].

**Figure 8:**
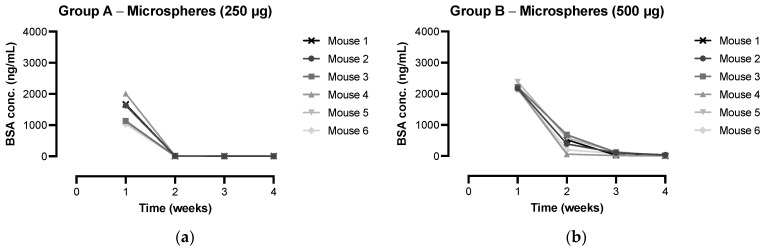

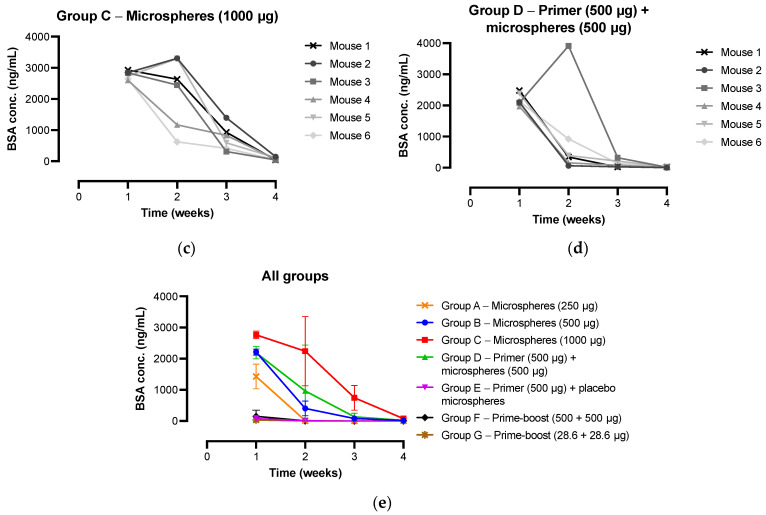
BSA levels in mouse plasma over time after immunization with different BSA formulations (group A to D). Mice (*n* = 6 per group) were immunized with: (**a**) 250 µg BSA-microspheres in CMC solution; (**b**) 500 µg BSA-microspheres in CMC solution; (**c**) 1000 µg BSA-microspheres in CMC solution; and (**d**) 500 µg BSA in PBS together with 500 µg BSA-microspheres in CMC solution. (**e**) The averages of the BSA levels measured in all mice were calculated for each group (group A to G) and presented for all groups together.

For most of the mice from the treatment groups (groups A to C), IgG antibody titers continued to increase up to four weeks after administration of the formulations, which can be considered an indirect indication of sustained release of BSA from the microspheres. After these four weeks, antibody titers hardly increased, which suggests that the release of BSA from the microspheres had ceased. These results are in line with the in vitro release data (Figure 4a), where the vast majority of the encapsulated BSA was released in a near-linear fashion over a period of four weeks. The development of antigen-specific antibodies, however, takes approximately one week [7,49]. An increase in IgG antibody titers up to four weeks, therefore, suggests an in vivo release duration of only three weeks. This indicates that the release of BSA was faster in vivo than in vitro, probably due to accelerated microsphere degradation in vivo, for instance, caused by increased liquid uptake into the polymer and foreign body responses [8,50,51]. Lipids and other biological molecules can act as plasticizers or affect the surface tension, which enhances water uptake. Moreover, free radicals, acidic products, or enzymes produced by macrophages that form around the microspheres can accelerate polymer degradation. To gain more insight into the in vivo pharmacokinetics of BSA, plasma samples from weeks 1 to 4 of groups A to G were analyzed for BSA levels as well (Figure 8). Sustained release of BSA from the microspheres into the systemic circulation was demonstrated, with plasma BSA concentrations being dependent on the administered dose, as expected. Peak plasma concentrations were 1429 ± 397, 2214 ± 99, and 2762 ± 127 ng BSA/mL for 250, 500, and 1000 µg BSA, respectively. For most of the mice receiving BSA-loaded microspheres, the highest plasma BSA concentration was measured one week after administration, followed by a strong decline in the concentration (Figure 8a–d). After four weeks, only low levels of BSA were still measured. In contrast, the highest release rate in vitro was reached after three weeks of incubation (Figure 5), which indicates that the release of BSA from the microspheres was indeed faster in vivo than in vitro. It is, however, also possible that the decline in BSA plasma concentration after one week was due to antibody formation, as was previously observed by van Dijk et al. after injection of sustained-release microspheres containing a human serum albumin construct [34]. Likewise, in our study, the induced antibodies might have formed a complex with BSA, which prevented the model antigen from binding to the capture antibodies of the ELISA and, thus, from being detected with the assay. Furthermore, the theoretical plasma BSA concentrations of weeks 2 to 4 can be calculated based on the actual plasma BSA concentrations of the previous week, assuming a BSA half-life of 1 day [52,53]. For almost all mice of groups B to D, the actual plasma BSA concentrations of weeks 2 to 4 were higher than the theoretical concentrations. This suggests that at least some release of BSA from the microspheres was still ongoing during these weeks.

Altogether, these findings demonstrate that single-injection microspheres providing a sustained release of BSA can induce strong humoral immune responses in mice, with antibody titers similar to the immune response induced by a prime-boost injection of BSA in PBS. Sustained-release microspheres, therefore, might be a viable alternative to the conventional prime-boost immunization schedule. Further research is, however, needed to determine whether the developed microspheres are also suitable for the delivery of a clinically relevant vaccine and which dose of antigen is optimal for strong antibody induction. In this study, relatively high doses of BSA were administered, while lower doses might have been sufficient as well. Once a clinically relevant antigen has been incorporated, IgG subclasses (e.g., IgG1 and IgG2a) and cellular immune responses could be determined in addition to total IgG. This will provide insight into qualitative aspects of the immune response induced by sustained-release microspheres. Moreover, tailoring the release duration to the specific needs of a vaccine is essential for the use of the sustained-release microspheres for a broad variety of vaccines. According to the in vitro release results, the release duration could be varied by varying the blend ratio of the polymers used but changing the composition of the polymers is an option as well. However, establishing an in vitro-in vivo correlation remains difficult, as there are many factors in play that affect the pharmacokinetics of an antigen. Examples are plasma clearance and antibody formation, but also lymphatic uptake and metabolism, interference of components of the s.c. extracellular matrix, and protein degradation at the injection site [34]. Determining the in vivo release or the plasma concentration as a surrogate indicator of release is, therefore, recommended.

## 4. Conclusions

Novel multi-block copolymers composed of amorphous, hydrophilic PCL-PEG-PCL blocks and semi-crystalline PDO blocks were used to produce sustained-release microspheres containing the model antigen BSA. The membrane emulsification method enabled the production of uniformly sized particles with the desired size and morphology and high EE. In vitro release studies showed that the release rate could be modulated by adjusting the blend ratio of the two multi-block copolymers. All formulations exhibited sustained release of BSA with low initial burst. Microspheres consisting of a 92.5:7.5 polymer blend released BSA in vitro in a near-linear fashion over a period of approximately four weeks, after which BSA continued to slowly diffuse out for another two weeks. We demonstrated that these microspheres were able to induce a strong BSA-specific IgG antibody response in vivo after s.c. administration in mice. The immune response was equal to that elicited by a prime-boost injection of BSA in PBS administered at 0 and 3 weeks, and the IgG titers followed the same pattern as the in vitro BSA release. Pharmacokinetic analysis of the microspheres demonstrated that in vivo release of BSA was probably ongoing up to at least four weeks as well, although peak plasma concentrations were already reached one week after administration and after four weeks only low levels of BSA were still detected. This suggests that the release of BSA was faster in vivo than in vitro, although the early decline in plasma BSA concentration might also have been caused by the formation and subsequent elimination of antigen–antibody complexes. Converting in vitro release and plasma concentration profiles into in vivo release profiles, thus, remains a challenge. This research shows the potential of sustained-release microspheres as an alternative to the conventional prime-boost immunization schedule. Ultimately, this technology could contribute to the development of single-injection vaccines and improvements in global vaccination coverage. Further studies with a clinically relevant antigen are, however, necessary to evaluate the clinical potential of the microspheres.

## Figures and Tables

**Table 1:** Characterization of the multi-block copolymers used in this study.

	Polymer A_1_	Polymer A_2_	Polymer B
	20[PCL-PEG_3000_-PCL]-80[PDO]		50[PCL-PEG_1000_-PCL]-20[PDO]
Molar caprolactate/PEG ratio (^1^H NMR)	6.8 (6.4 in-weight)	6.4 (6.4 in-weight)	8.3 (8.6 in-weight)
Molar dioxanonate/PEG ratio (^1^H NMR)	141.6 (147.6 in-weight)	155.2 (147.5 in-weight)	18.5 (18.9 in-weight)
Weight ratio PCL-PEG-PCL/PDO block (^1^H NMR)	21.2/78.8	19.6/80.4	49.9/50.1
M_n_ (×10^4^ g/mol)	2.8	1.5	3.6
M_w_ (×10^4^ g/mol)	4.3	4.5	6.7
Intrinsic viscosity (dL/g)	0.70	0.69	0.73
1,4-dioxane content (ppm)	<18	<18	<18
T_g_ (°C)	−15	−14	−57 and −23
T_m_ (°C)	34 and 88	34 and 89	88

**Table 2:** Experimental parameters and settings of different bovine serum albumin (BSA)-loaded and placebo microsphere formulations.

Formulation Parameters		Formulation					
		A	B	C	D	E	F
Weight ratio polymer A:polymer B ^1^		100:0	92.5:7.5	85:15	75:25	50:50	92.5:7.5
Target BSA loading (wt-%)		5	5	5	5	5	n.a. ^2^
Batch size (g)		1.5	3.5	3.5	3.5	1.5	3.5
Ultra-Turrax^®^	Speed (rpm)	21,000	25,000	25,000	25,000	21,000	25,000
	Time (s)	40	60	60	60	40	60
Extraction	Vessel size (L)	2	5	5	5	2	5
	Stirrer type	Anchor-type stirring shaft	Stirring bar (10.8 × 2.6 cm)	Stirring bar (10.8 × 2.6 cm)	Stirring bar (10.8 × 2.6 cm)	Anchor-type stirring shaft	Stirring bar (10.8 × 2.6 cm)
	Stirrer speed (rpm)	200	75	75	75	200	75
	Airflow (L/min)	5	10	10	10	5	10
	Time (h)	3	4	4	4	3	4

^1^ Polymer A_1_ was used for the preparation of formulation A and E; polymer A_2_ was used for formulation B, C, D, and F. ^2^ Formulation F consisted of placebo microspheres that did not contain any BSA.

**Table 3:** Theoretical PEG, PCL, PDO, BDO, and BDI content of microspheres prepared from different weight ratios of polymer A and B.

Ratio Polymer A: Polymer B	Total PEG (wt-%)	PEG_3000_ (wt-%)	PEG_1000_ (wt-%)	PCL (wt-%)	PDO (wt-%)	BDO (wt-%)	BDI (wt-%)
100:0	15	15	0	4	73	3	5
92.5:7.5	15.675	13.875	1.8	5.425	70.975	2.925	5
85:15	16.35	12.75	3.6	6.85	68.95	2.85	5
75:25	17.25	11.25	6	8.75	66.25	2.75	5
50:50	19.5	7.5	12	13.5	59.5	2.5	5

**Table 4:** Overview of the groups and the corresponding formulations used for the in vivo immunization study in mice.

Group	Type of Group	Formulation Composition	Administration	Total Dose	Average Daily Dose	Week of Administration	Number of Animals
A	Treatment	BSA-MSP in CMC solution ^1^	7.28 mg MSP-F in 193 μL CMC solution ^1^	250 μg	7.1 μg ^2^	0	6
B	Treatment	BSA-MSP in CMC solution	14.6 mg MSP-F in 187 μL CMC solution	500 μg	14.3 μg ^2^	0	6
C	Treatment	BSA-MSP in CMC solution	29.2 mg MSP-F in 174 μL CMC solution	1000 μg	28.6 μg ^2^	0	6
D	Positive control/ treatment	BSA in PBS + BSA-MSP in CMC solution	500 μg BSA in 100 μL PBS + 14.6 mg MSP-B in 87 μL CMC solution ^1^	1000 μg (500 μg + 500 μg)	514.3 μg on day 1, 14.3 μg for remaining days ^2^	0	6
E	Positive control/placebo	BSA in PBS + placebo MSP in CMC solution	500 μg BSA in 100 μL PBS + 14.6 mg MSP-F in 87 μL CMC solution	500 μg	500 μg on day 1	0	6
F	Positive control	BSA in PBS (prime-boost)	500 μg BSA in 200 μL PBS (at 0 and 3 weeks)	1000 μg (500 μg + 500 μg)	500 μg on day 1, 500 μg on day 22	0 and 3	6
G	Positive control	BSA in PBS (prime-boost)	28.6 μg BSA in 200 μL PBS (at 0 and 3 weeks)	57.1 μg (28.6 μg + 28.6 μg)	28.6 μg on day 1, 28.6 μg on day 22	0 and 3	6
H	Negative control	PBS	200 μL PBS (at 0 and 3 weeks)	-	-	0 and 3	3
I	Negative control	CMC solution	200 μL CMC solution	-	-	0	3

^1^ MSP = microspheres, MSP-B = microspheres of formulation B, MSP-F = microspheres of formulation F. ^2^ Assuming an in vivo release duration of five weeks.

**Table 5:** Characteristics of BSA-loaded and placebo microsphere formulations prepared with different polymer blend ratios ^1^.

Formulation	Ratio Polymer A: Polymer B	d10 (µm)	d50 (µm)	d90 (µm)	CV (%)	Actual Loading (wt-%)	EE (%)
A	100:0	30.4	38.7	50.5	21.0	4.4 ± 0.1	87.4 ± 1.0
B	92.5:7.5	30.8	39.9	52.4	21.8	4.5 ± 0.5	89.9 ± 10.0
C	85:15	30.6	39.5	51.5	21.7	4.9 ± 0.3	97.3 ± 5.5
D	75:25	30.5	39.2	51.4	21.9	4.9 ± 0.2	96.9 ± 4.5
E	50:50	29.9	39.0	51.6	24.5	5.1 ± 1.3	101.7 ± 26.4
F	92.5:7.5	31.2	42.7	58.2	27.4	n.a. ^2^	n.a. ^2^

^1^ Blend ratio 92.5:7.5 (in grey) was selected for the in vivo proof-of-concept study in mice. ^2^ Formulation F consisted of placebo microspheres that did not contain any BSA.

## Data Availability

Not applicable.

## Supplementary Materials

The following supporting information can be downloaded at: https://www.mdpi.com/article/10.3390/pharmaceutics15020676/s1. Figure S1: BSA-specific IgG antibody titers in mouse plasma over time after immunization with different BSA formulations (group A to G). Mice (*n* = 6 per group) were immunized with (a) 250 µg BSA-microspheres in carboxymethyl cellulose (CMC) solution; (b) 500 µg BSA-microspheres in CMC solution; (c) 1000 µg BSA-microspheres in CMC solution; (d) 500 µg BSA in phosphate-buffered saline (PBS) together with 500 µg BSA-microspheres in CMC solution; (e) 500 µg BSA in PBS together with placebo microspheres in CMC solution; (f) 500 + 500 µg BSA in PBS, prime injection (week 0) and booster injection (week 3); and (g) 28.6 + 28.6 µg BSA in PBS, prime injection (week 0) and booster injection (week 3). The dotted lines represent the cut-off value for the IgG antibody titer, i.e., a titer of 6.64 log_2_, corresponding to the starting dilution of the plasma samples of 1:100. Values below this titer could not be measured. Samples with a reading for the least diluted plasma (i.e., 100× diluted) lower than the cut-off value were assigned an IgG antibody titer of 5.64 log_2_, corresponding to a dilution of 1:50, which would be one dilution below the starting dilution. For these samples, dashed lines were used to connect the data point below the cut-off value with the next time point. The negative control groups receiving PBS (group H) and CMC solution (group I) are not presented in this figure. Figure S2: Area under the IgG titer–time curve (AUC) values of the BSA-specific IgG antibody titer vs. time graph (Figure S1). Statistical comparisons between the mice of the different groups were performed using the ordinary ANOVA, followed by Tukey’s multiple comparisons test (* *p* < 0.05, ** *p* < 0.01). For clarity reasons, statistical comparison is only indicated where *p* < 0.05 (*) or *p* < 0.01 (**), and differences for all other comparisons were non-significant. The negative control groups receiving PBS (group H) and CMC solution (group I) are not presented in this figure. MSP = microspheres.
