# Supplementary material for: A Single Injection with Sustained-Release Microspheres and a Prime-Boost Injection of Bovine Serum Albumin Elicit the Same IgG Antibody Response in Mice
Source: Pharmaceutics. 2023 Feb 16;15(2):676. doi: 10.3390/pharmaceutics15020676 (PMC9960585; doi:10.3390/pharmaceutics15020676)
Supplement: Supplementary file 1 [file pharmaceutics-15-00676-s001.zip › pharmaceutics-2203838-supplementary.pdf]

pharmaceutics

MDPI

Supplementary Materials: A single injection with sustained-release microspheres and a prime-boost injection of bovine serum albumin elicit the same IgG antibody response in mice

Renée S. van der Kooij, Martin Beukema, Anke L. W. Huckriede, Johan Zuidema, Rob Steendam, Henderik W. Frijlink and Wouter L. J. Hinrichs

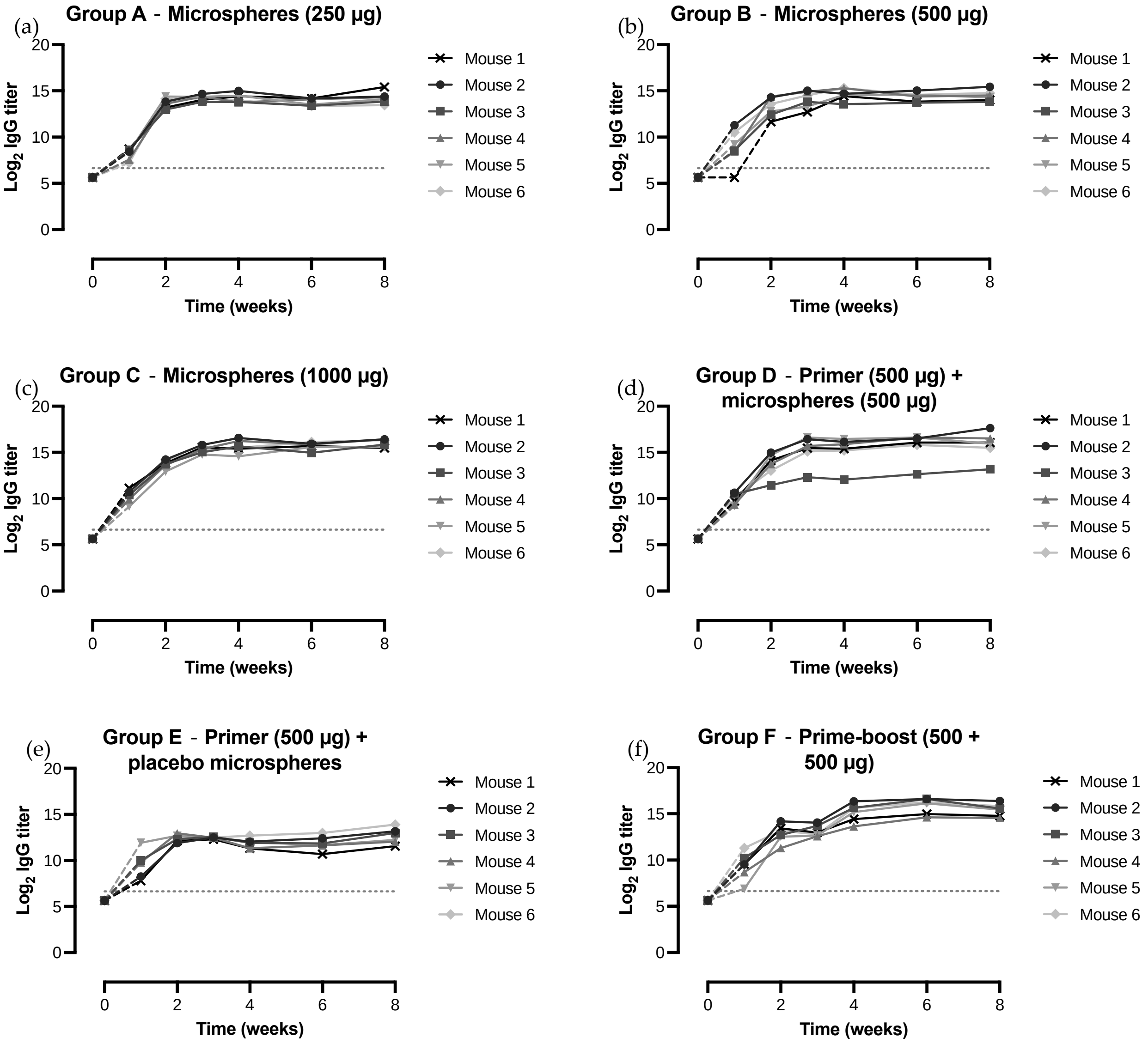

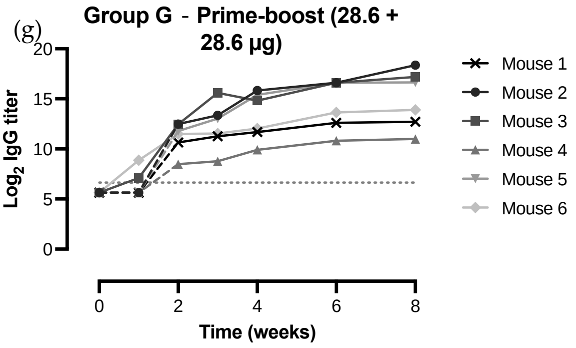

Figure S1. BSA-specific IgG antibody titers in mouse plasma over time after immunization with different BSA formulations (group A to G). Mice (n = 6 per group) were immunized with: (**a**) 250 µg BSA-microspheres in carboxymethyl cellulose (CMC) solution; (**b**) 500 µg BSA-microspheres in CMC solution; (**c**) 1000 µg BSA-microspheres in CMC solution; (**d**) 500 µg BSA in phosphate-buffered saline (PBS) together with 500 µg BSA-microspheres in CMC solution; (**e**) 500 µg BSA in PBS together with placebo microspheres in CMC solution; (**f**) 500 + 500 µg BSA in PBS, prime injection (week 0) and booster injection (week 3); and (**g**) 28.6 + 28.6 µg BSA in PBS, prime injection (week 0) and booster injection (week 3). The dotted lines represent the cut-off value for the IgG antibody titer, *i.e.* a titer of 6.64 log_2, corresponding to the starting dilution of the plasma samples of 1:100. Values below this titer could not be measured. Samples with a reading for the least diluted plasma (*i.e.* 100x diluted) lower than the cut-off value were assigned an IgG antibody titer of 5.64 log_2, corresponding to a dilution of 1:50, which would be one dilution below the starting dilution. For these samples, dashed lines were used to connect the data point below the cut-off value with the next time point. The negative control groups receiving PBS (group H) and CMC solution (group I) are not presented in this figure.

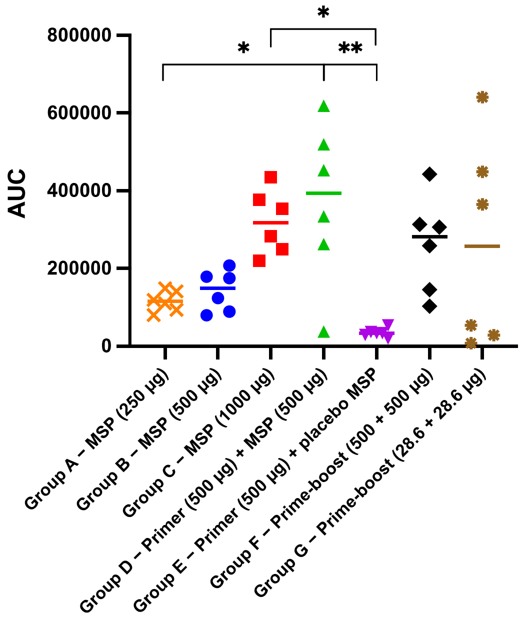

Figure S2. Area under the IgG titer–time curve (AUC) values of the BSA-specific IgG antibody titer vs. time graph (Figure S1). Statistical comparisons between the mice of the different groups were performed using the ordinary ANOVA, followed by Tukey's multiple comparisons test (* $p < 0.05$, ** $p < 0.01$). For clarity reasons, statistical comparison is only indicated where $p < 0.05$ (*) or $p < 0.01$ (**), and differences for all other comparisons were non-significant. The negative control groups receiving PBS (group H) and CMC solution (group I) are not presented in this figure. MSP = microspheres.
